# Development and validation of a bioanalytical method for the quantification of the CDK4/6 inhibitors abemaciclib, palbociclib, and ribociclib in human and mouse matrices using liquid chromatography-tandem mass spectrometry

**DOI:** 10.1007/s00216-019-01932-w

**Published:** 2019-06-17

**Authors:** Alejandra Martínez-Chávez, Hilde Rosing, Michel Hillebrand, Matthijs Tibben, Alfred H. Schinkel, Jos H. Beijnen

**Affiliations:** 1 Department of Pharmacy & Pharmacology, The Netherlands Cancer Institute, Plesmanlaan 121, 1066 CX Amsterdam, The Netherlands; 2 Division of Pharmacology, The Netherlands Cancer Institute, Plesmanlaan 121, 1066 CX Amsterdam, The Netherlands; 3 Division of Pharmacoepidemiology and Clinical Pharmacology, Utrecht Institute for Pharmaceutical Sciences, Utrecht University, David de Wied building, Universiteitsweg 99, 3584 CG Utrecht, The Netherlands

**Keywords:** Abemaciclib, Palbociclib, Ribociclib, LC-MS/MS, Plasma, Tissue homogenates

## Abstract

A novel method was developed and validated for the quantification of the three approved CDK4/6 inhibitors (abemaciclib, palbociclib, and ribociclib) in both human and mouse plasma and mouse tissue homogenates (liver, kidney, spleen, brain, and small intestine) using liquid chromatography coupled to tandem mass spectrometry (LC-MS/MS). For all matrices, pretreatment was performed using 50 μL of sample by protein precipitation with acetonitrile, followed by dilution of the supernatant. Chromatographic separation of the analytes was done on a C18 column using gradient elution. A full validation was performed for human plasma, while a partial validation was executed for mouse plasma and mouse tissue homogenates. The method was linear in the calibration range from 2 to 200 ng/mL, with a correlation coefficient (*r*) ≥0.996 for each analyte. For both human and mouse plasma, the accuracy and precision were within ±15% and ≤15%, respectively, for all concentrations, except for the lower limit of quantification, where they were within ±20% and ≤20%, respectively. A fit-for-purpose strategy was followed for tissue homogenates, and the accuracy and precision were within ±20% and ≤20%, respectively, for all concentrations. Stability of all analytes in all matrices at different processing and storage conditions was tested; ribociclib and palbociclib were unstable in most tissue homogenates and conditions were modified to increase the stability. The method was successfully applied for the analysis of mouse samples from preclinical studies. A new ribociclib metabolite was detected in mouse plasma samples with the same *m/z* transition as the parent drug.

**Electronic supplementary material:**

The online version of this article (10.1007/s00216-019-01932-w) contains supplementary material, which is available to authorized users.

## Introduction

The development of new small molecules for targeted therapy in cancer has been accelerated over the past years with the progress of high-resolution molecular techniques [1]. Since cancer cells present a dysregulated cell cycle, the cyclin-dependent kinases (CDKs), enzymes that play a key role in cell cycle control, have been regarded as promising targets for new anticancer molecules [2, 3]. The first generation of CDK inhibitors failed in clinical studies because they were found to be toxic to non-cancerous cells due to their lack of selectivity. In contrast, the second generation of this class of compounds, which is selective for CDK4/6, has shown efficacy in the clinic [2]. So far, three molecules within this class have been approved by the U.S. Food and Drug Administration (FDA): palbociclib in 2015 and ribociclib and abemaciclib both in 2017. These drugs specifically inhibit the CDK 4/6-cyclin D complex, thereby preventing the inactivation of the retinoblastoma (Rb) tumor suppressor protein, and arresting the cell cycle between G-phase and S-phase [3]. Despite these drugs interacting with their targets through the same 2-aminopyrimidine group, they differ in selectivity and potency [4]. Palbociclib and ribociclib are highly selective for CDK4/6 compared with other CDKs, and abemaciclib shows the highest potency with an IC_50_ for CDK4/CDK6 of 2/5 nM, compared with 9–11/15 and 10/39 nM for palbociclib and ribociclib, respectively [4, 5]. This is related to differences in their molecular structures and drug-target binding. Abemaciclib presents two fluorine atoms around the ATP-binding pocket, whereas ribociclib and palbociclib contain much larger substituents (a dimethylamino group, and metlhylketone and adjacent methyl groups, respectively) which might influence their increase in selectivity. Furthermore, the lipophilicity (cLogP) of each compound correlates with their potency; the cLogP of abemaciclib (5.5) is significantly higher than that of palbociclib (2.7) or ribociclib (2.3) [4]. These molecules are currently indicated to treat certain types of breast cancer in combination with hormone therapy. Furthermore, the efficacy of each compound to treat other cancer types is still under investigation in preclinical models and clinical trials, either as single agents or in combination with other drugs [6, 7].

Validated bioanalytical methods are needed to support these investigations. To date, some bioanalytical methods have been reported for the quantification of palbociclib in biological matrices. Nguyen et al. reported a validated quantitative analysis for palbociclib in breast tumor homogenates using LC-MS/MS [8]. Smith et al. introduced the use of an automated micro-sample processing for the pretreatment of mouse plasma samples prior to LC-MS/MS analysis of palbociclib; however, the availability of this system is restricted in many laboratories [9]. Recently, Paul et al. described two bioanalytical methods for the quantification of palbociclib in rat plasma using an LC-MS method using a high-resolution quadrupole time of flight (Q-TOF) mass spectrometer, which is more convenient for qualitative rather than quantitative assays; therefore, the instrumentation is restricted in most bioanalytical laboratories [10, 11]. For ribociclib, two bioanalytical assays have been published. A method for the quantification of ribociclib in mouse plasma and Ringer’s solution for a cerebral microdialysis study was first described [12]. Recently, Bao et al. reported a method for the quantification of ribociclib in human plasma, brain tumor, and CSF samples [13]. Finally, Raub et al. reported the use of LC-MS/MS for the quantification of palbociclib and abemaciclib in biological matrices, but no further details of the methods and their performance were described [14]. Since our particular interest is to characterize the function of transporters and metabolic enzymes in drug pharmacokinetics and tissue distribution, a validated assay is needed for the quantification of these compounds in mouse plasma and tissue homogenates. Previously published assays for the determination of abemaciclib, palbociclib, and ribociclib do not include all the matrices needed for our study. Since these compounds show similar molecular structures, one versatile method could be used to optimize bioanalytical measurements of samples from different preclinical studies or patients, using one single method and thus simplifying the logistics of the bioanalytical laboratory.

Therefore, the objective of this study was to develop and validate the first LC-MS/MS method for the quantitative analysis of the three approved CDK4/6 inhibitors abemaciclib, palbociclib, and ribociclib in human and mouse plasma and mouse tissue homogenates, including the liver, kidney, spleen, brain, and small intestine. A full validation was performed in human plasma, while for mouse plasma and tissue homogenates, a partial validation was executed. Calibration standards in a surrogate matrix (human plasma) were used for the quantification in these matrices. This method was set up to support pharmacokinetics and tissue distribution studies in mice.

## Materials and methods

### Chemicals

Abemaciclib, palbociclib, and ribociclib, as well as their internal standards (IS) ^2^H_8_-abemaciclib, ^2^H_8_-palbociclib, and ^2^H_6_-ribociclib (100.0% purity for all compounds), were purchased from Alsachim (Illkirch-Graffenstaden, France). The isotopic enrichment of the internal standards was 98.0%, 98.3%, and 99.9% for ^2^H_8_-abemaciclib, ^2^H_8_-palbociclib, and ^2^H_6_-ribociclib, respectively. Methanol (MeOH), acetonitrile (ACN), formic acid, and water (all supragradient grade) were supplied by Biosolve Ltd (Valkenswaard, The Netherlands). Dimethylsulfoxide (DMSO, SeccoSolv ≥ 99.9% purity) was obtained from Merck (Darmstadt, Germany), and ammonium bicarbonate (LC-MS grade) was from Sigma-Aldrich (Darmstadt, Germany).

### Blank matrices

Control human K_2_EDTA plasma originated from Bioreclamations LLC (Hicksville, NY, USA). Mouse sodium heparin plasma and mouse organs (including the brain, kidneys, liver, small intestine, and spleen) were obtained from the animal laboratory of the Netherlands Cancer Institute (Amsterdam, The Netherlands). All blanks were stored at − 20 °C prior to use.

#### Tissue homogenate preparation

Blank tissue homogenates were prepared using 6.5 mm ceramic beads and a Fast Prep-24™ 5G (MP Biomedicals Inc., Santa Ana, CA, USA). Bovine serum albumin (BSA, Fraction V), obtained from Roche Diagnostics GmbH (Mannheim, Germany) was dissolved in distilled water (B Braun Medical, Melsungen, Germany), at a concentration of 4% (*w*/*v*). After weighing the complete organ, a volume of 3 mL of this solution was added to the liver and small intestine, 2 mL to the kidneys, and 1 mL to the brain and spleen. The tissue/BSA ratio (*w*/*v*) of the homogenates were approximately 0.4:1 for the brain and liver, 0.1:1 for the spleen, 0.2:1 for the kidneys, and 0.25:1 for the small intestine. All samples were shaken in the Fast Prep at 6.0 m/s during 60 s.

### Stock and working solutions

Two independent stock solutions (one for calibration standards and the other for quality control (QC) samples) of each analyte were prepared at 1 mg/mL in DMSO for abemaciclib and ribociclib, and in 0.1% formic acid in water for palbociclib. All stock solutions for the analytes were stable for at least 12 months. Individual stock solutions of each IS were prepared at 1 mg/mL in the same solvents as their corresponding analyte.

From the stock solutions, six working solutions were prepared for calibration standards adding all analytes at a concentration of 40, 200, 1000, 2000, 3000, and 4000 ng/mL in MeOH. For the QC samples, four working solutions were prepared at 40, 100, 2000, and 3000 ng/mL. The IS-working solution was prepared by mixing and diluting all the IS stock to obtain 250 ng/mL in MeOH. Stock and working solutions were stored at − 20 °C.

### Calibration standards and QC samples

Analyte working solutions were diluted 1:20 (*v*/*v*) with blank human plasma to obtain six calibration standards with final concentrations of 2, 10, 50, 100, 150, and 200 ng/mL for all analytes.

The same dilution was used to prepare QC samples in human plasma, mouse plasma, and mouse tissue homogenates at concentrations of 2 (lower limit of quantification (LLOQ)), 5 (QC L), 50 (QC M), and 150 ng/mL (QC H) for each analyte.

### Sample pretreatment

50 μL of the sample (either from a preclinical study, calibration standard, or QC) was mixed with 25 μL of IS-working solution, followed by 150 μL of ACN for protein precipitation. Samples were vortex mixed and centrifuged at 23,100*g* for 10 min at 20 °C. An aliquot of 80 μL of supernatant was diluted with 120 μL of 10 mM ammonium bicarbonate in water:MeOH (1:1 *v*/*v*). The final extract was transferred to a glass vial with insert, and 10 μL was injected to the LC-MS/MS system for analysis.

### LC-MS/MS system and method

The chromatographic system consisted of an Agilent 1100 chromatograph (Palo Alto, CA, USA) equipped with a binary pump (Model G1312A), a degasser (Model G1379A), an autosampler (Model G1367A), and a column oven (Model G1316A). The analytes were separated using a Gemini C18 column (50 × 2.0 mm ID, 5 μm) coupled to a Gemini C18 (4 × 2 mm) guard column (Phenomenex, Torrance, CA, USA), and a mobile phase composed of 10 mM ammonium bicarbonate in water (eluent A) and 10 mM ammonium bicarbonate in water-methanol (1:9, *v*/*v*; eluent B). Gradient elution was applied and the eluent composition and flow rate are described in Table 1. The temperature of the autosampler and column oven was set at 6 °C and 40 °C, respectively.

**Table 1:** Gradient and flowrate settings

Time (min)	B%	Flow rate (μL/min)
Initial	55	250
1.0	100	400
2.0	100	400
2.5	55	400
3.0	55	400
4.0	100	500
4.5	100	500
5.5	55	500
6.5	55	250
7.0	55	250

For detection, an API 3000 triple quadrupole with electrospray ionization (ESI) mass spectrometer (Sciex, Foster City, CA, USA) was operated in a positive ion mode. Samples were acquired via multiple reaction monitoring (MRM) and analyzed using the Analyst software version 1.6.2 (Sciex). The following general settings were used to detect all the compounds: the ion spray voltage was set at 5000 V, the temperature for the solvent evaporation was established at 350 °C, and the nebulizer, curtain, and collision gases were set at 3, 9, and 11 (arbitrary units), respectively. The entrance and focusing potentials were 10 and 350 V, respectively, and the scan/dwell time was 100 ms. The analyte-specific settings are described in Table 2. The chromatographic system was coupled to the mass spectrometer by a divert valve, which directed the flow to the detector from 1 to 3.5 min and the rest to the waste container.

**Table 2:** MS compound specific parameters

	Abemaciclib	Palbociclib	Ribociclib
Analyte parent to product transition (*m*/*z*)	507 → 393	448 → 380	435 → 322
IS parent to product transition (*m*/*z*)	515 → 393	456 → 388	441 → 322
Declustering potential (V)	56	71	71
Collision energy (V)	29	39	47
Collision cell exit potential (V)	26	24	30

### Method validation

A full method validation was performed in human plasma and was based on the U.S. FDA and European Medicine Agency (EMA) guidelines for bioanalytical method validation [15, 16]. A partial validation was executed for mouse plasma and the following performance characteristics were tested: accuracy and precision, selectivity, dilution integrity, and stability. Finally, for tissue homogenates, a fit-for-purpose strategy was followed according to Xue et al. [17], where accuracy and precision, selectivity, dilution integrity, and stability were evaluated using the acceptance criteria for method qualification.

#### Calibration model

Human plasma calibration standards (6 non-zero standards with duplicate points at each concentration in the range 2 to 200 ng/mL for each analyte), including a blank and a zero calibration standard (blank spiked with IS), were freshly prepared in duplicate and analyzed at the beginning and the end of each analytical run. A linear model with a 1 ∕ *x*^2^ weighting factor was used to describe the concentration-response relationship for all analytes, where *x* is the analyte concentration. At least 75% of non-zero calibration standards should meet the following criteria: their calculated concentrations should be within ± 15% of the nominal concentrations, except at LLOQ where the calculated concentration should be ± 20% of the nominal concentration in a minimum of three validation runs.

#### Selectivity and specificity

The selectivity of the method was established by the analysis of LLOQ and blank samples from 6 different batches of control human K_2_EDTA and mouse plasma. For each tissue homogenate, one batch was evaluated. LC-MS/MS chromatograms of the blanks and LLOQ samples were monitored and compared for chromatographic integrity and potential interferences.

Furthermore, the cross analyte/internal standard interferences were determined by separately spiking abemaciclib, palbociclib, and ribociclib to control human plasma at the upper limit of quantification (ULOQ). Independently, blank human plasma was spiked also with each internal standard at the concentration used in the assay. For each sample, any interference at the retention times of the analytes and internal standard was evaluated.

In at least 4 of 6 batches, the response of the interfering peaks at the retention times of the analytes should be ≤ 20% of the LLOQ response at the LLOQ, and for the interfering peaks at the retention time of the internal standard, their response should be ≤ 5% of the response of the internal standard. LLOQ samples should be within ± 20% of the nominal concentration.

#### Lower limit of quantification

This parameter was evaluated comparing the response of the zero calibrator and the LLOQ in three validation runs. To meet the acceptance criteria, the response at the LLOQ should be at least 5 times the response compared with the zero calibrator response for each CDK4/6 inhibitor.

#### Carryover

Carryover was tested in three analytical runs by injecting two blank matrix samples after the ULOQ. The percentage of response compared to the LLOQ obtained for each analyte in the blank matrix samples was calculated. Carryover should not exceed 20% of LLOQ.

#### Accuracy and precision

QC samples were prepared in human and mouse plasma and mouse tissue homogenates at the concentrations described in the “Calibration standards and QC samples” section. Five replicates of each level were analyzed in three analytical runs for human plasma. For the remaining matrices, five replicates of each level were tested in one analytical run. The intra-assay coefficient of variation (CV) and bias (between the nominal and measured concentrations) were calculated for the precision and the accuracy, respectively. Furthermore, for human plasma, the inter-assay CV (calculated by ANOVA) and bias were determined. For plasma matrices, the accuracy should be within ± 15% of nominal concentrations, and for the precision, the CV should be ≤ 15% for all concentration levels, except at LLOQ, where ± 20% and ≤ 20%, respectively, are accepted. For the accuracy and precision in tissue homogenates, ± 20% and ≤ 20% were accepted at all concentration levels, respectively.

#### Matrix factor and recovery

Matrix effects were investigated in 6 different batches of human plasma at QC L and QC H concentrations. Each concentration level was prepared in the presence of matrix (each blank plasma batch was processed until final extract and spiked with the corresponding QC working solution) and in the absence of matrix (QC working solutions diluted with organic solvents). The matrix factor (MF) was determined for each lot of matrix by calculating the ratio of the peak area in the presence of matrix to the peak area in the absence of matrix. Furthermore, the IS-normalized MF was calculated dividing the MF of the analyte by the MF of the IS.

For the recovery, the processed QC L and QC H samples were compared with the matrix-absent samples (previously described) and the percentage of recovery was calculated as well as the CV for each concentration level. The CV for the matrix factor and the recovery should be < 15%.

#### Dilution integrity

The integrity of mouse plasma and tissue homogenate samples diluted with control human plasma was investigated. Five replicates of each homogenate at around 5 times the ULOQ (1000 ng/mL) were prepared and diluted 10 times with control human plasma. For mouse plasma dilution integrity, two samples were prepared in 5-fold at 25 and 1000 ng/mL and separately diluted 5 times in control human plasma. The accuracy and precision for these samples were calculated, and for the acceptance criteria, they should be within ± 15% of the nominal concentration and the CV should be ≤ 15%, respectively.

#### Stability

The stability of each analyte was investigated in all the previously mentioned matrices. For plasma (both human and mouse), the stability was determined in triplicate at two concentration levels, QC L and QC H, whereas for mouse tissue homogenates only at QC M (except the brain in one stability condition). For all matrices, the following stability conditions were assessed: short term at room temperature (RT), long term at − 20 °C, and after 3 freeze-thaw (F/T) cycles at − 20 °C/RT. Furthermore, for tissue homogenates, the short-term stability in ice-water, long-term stability at − 70 °C, and F/T stability at − 70 °C/ice-water were also evaluated. Additionally, the stability in the final extract was tested. For this, the QC L and QC H final extracts from the plasma, brain, and spleen and the QC M final extract from the liver, kidney and small intestine were stored at 4–8 °C up to 3 days. Lastly, the stability of the stock solutions at − 20 °C was investigated. Analytes were considered stable if the accuracy was ± 15% and ± 20% of nominal concentration for plasma and tissue homogenates, respectively.

### Preclinical application of the method

This method was developed to support pharmacokinetic and tissue distribution studies of the CDK4/6 inhibitors in mice (FVB background). These studies were done separately for each drug. Animal housing and studies were conducted according to institutional guidelines complying with Dutch and European Union legislation (approval number from The Dutch Central Animal Testing Committee: AVD301002016595). After a minimum of 2-h fasting, the drug solution was orally administered at doses of 10 mg/kg for abemaciclib (*n* = 6) and palbociclib (*n* = 4), and at 20 mg/kg for ribociclib (*n* = 6). Approximately 50 μL of blood was collected from the tip of the tail at several time points, except at the last one, when 500–1000 μL of blood was collected by cardiac puncture under isoflurane anesthesia. For the ribociclib pharmacokinetic study, the sampling time points were 0.25, 0.5, 1, 3, 8, and 24 h. Mice were sacrificed by cervical dislocation and the organs were collected and weighed. Plasma was obtained using sodium heparin as anticoagulant after centrifugation at 9000*g*, 4 °C, for 6 min. When necessary, 10 μL of mouse plasma was diluted with 40 μL of human plasma. The tissue homogenates were prepared according to the procedure described in the “Tissue homogenate preparation” section. Plasma and tissue homogenate samples were stored at − 20 and − 70 °C, respectively, until the LC-MS/MS analysis was performed.

### Qualitative analysis using HR-MS

High-resolution mass spectrometry (HR-MS) measurements were performed in an LC (Shimadzu, Kyoto, Japan) composed by an LC-20AD pump, a CTO-LOAC column oven, and a SIL-HTC autosampler, and coupled to an LTQ Orbitrap Discovery (Thermo Fisher Scientific, Waltham, MA, USA). A mouse plasma sample obtained after the pharmacokinetics study was injected into the system using the same chromatographic settings as described above, except for the gradient. In the initial conditions, the flow rate and the eluent B were set at 0.25 mL/min and 55%, respectively. These conditions were maintained for 2.5 min, % B increased up to 100% in 1 min, and it was held during 2.5 min. The gradient reverted back to the initial condition at 55% B in 0.1 min and held up to 8.0 min of the total run time.

## Results and discussion

### Method development

#### Mass spectrometry and chromatography

**Fig. 1:**
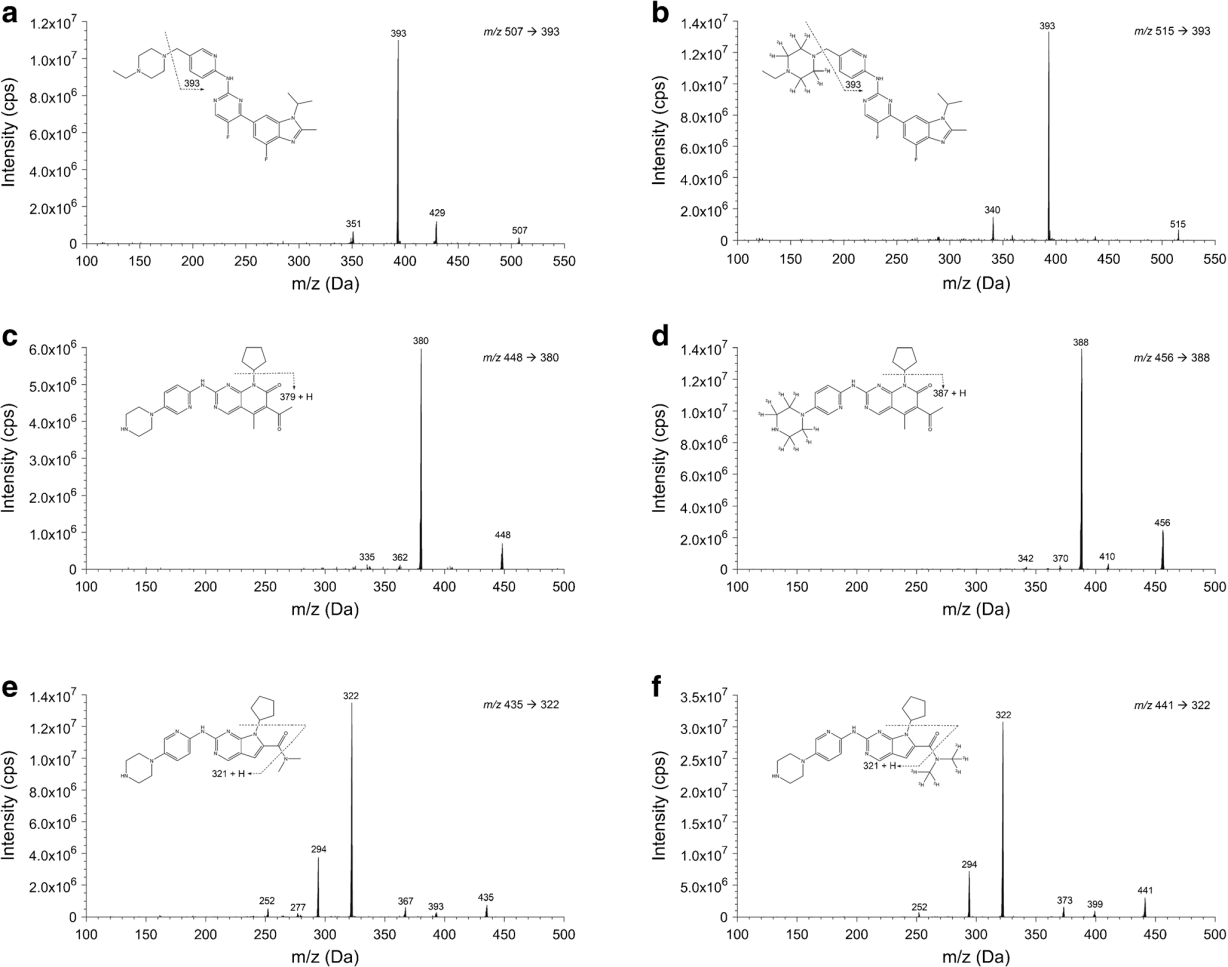
Mass spectra with the molecular structure and proposed MS fragmentation pattern of abemaciclib (**a**), palbociclib (**c**), ribociclib (**e**), and their stable isotopically labeled internal standards (**b**, **d**, **f**, respectively). The figure was created using ChemDraw Professional 15.0 and MATLAB R2017a software using the output from Sciex Analyst software

To establish the analyte-dependent parameter settings of the mass spectrometer, each compound (dissolved in MeOH:water (80:20 *v*/*v*) 1% formic acid) was directly infused into the MS at a concentration of 100 ng/mL for abemaciclib and palbociclib, and 1000 ng/mL for ribociclib. The most abundant fragment ion of each analyte was chosen for quantification (Fig. 1). A compound optimization was performed for each analyte and those values that produced the highest intensity in the parent ion response from each parameter were selected. The general settings were chosen by a flow injection analysis of ribociclib, to enhance the response intensity, since this compound showed the lowest intensity compared with palbociclib and abemaciclib.

Similarly to previous publications [8, 12], carryover post-injection was observed for all the CDK4/6 inhibitors. In general, the more hydrophobic the compound, the higher percentage of carryover was detected. Thus, abemaciclib was the compound that showed the highest carryover, followed by palbociclib and then ribociclib. In order to decrease the percentage of carryover, a washing step in the solvent gradient was included after the elution of all the analytes, and a narrow concentration range was chosen, from 2 to 200 ng/mL.

#### Sample pretreatment

Two different precipitation agents (MeOH:ACN (1:1, *v*/*v*) and ACN) and three different dilution solvents for the supernatant (acidic (formic acid 0.1% *v*/*v*), alkaline (10 mM ammonium bicarbonate in water:MeOH (1:1, *v*/*v*)), and water) were tested. The analyte responses were evaluated using all combinations of precipitation agents with dilution solvents. ACN showed the highest response for abemaciclib, whereas for the other two analytes, no difference was observed between both precipitation agents. The alkaline solvent was chosen to dilute the supernatant before injection, since it was observed that the percentage of carryover decreased for all analytes.

### Method validation

#### Calibration curve

**Fig. 2:**
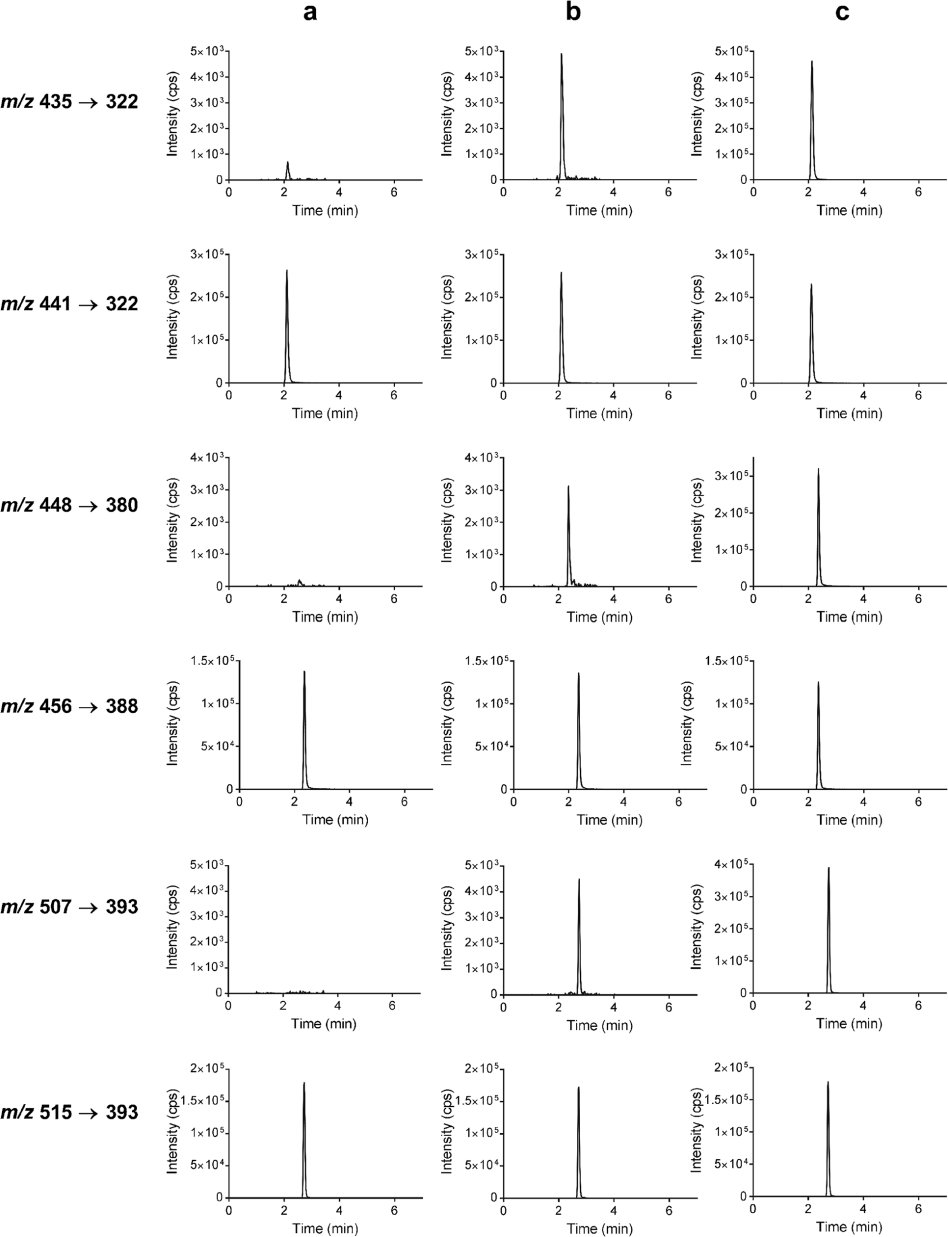
Representative chromatograms of control human plasma spiked with internal standards (**a**), spiked at the LLOQ (**b**), and spiked at the ULOQ (**c**) at the mass transitions of ribociclib (***m/z*435→322**), ribociclib-IS (***m/z*441→322**), palbociclib (***m/z*448→380**), palbociclib-IS (***m/z*456→388**), abemaciclib (***m/z*507→393**), and abemaciclib-IS (***m/z*515→393**). *Y*-axis in chromatograms of **a** and **b** series for the analyte mass transitions (***m/z*435→322**, ***m/z*448→380**, and ***m/z*507→393**) have the same scale, but *Y*-axis scale in chromatograms from **c** are adjusted to the highest response. The figure was created using the output form Sciex Analyst software along with GraphPad Prism 7 software

A linear model with a 1 ∕ *x*^2^ weighting factor properly described the concentration-response relationship for all the analytes. Representative chromatograms of each CDK 4/6 inhibitor and of their internal standards at the LLOQ and ULOQ are shown in Fig. 2. The correlation coefficients obtained (*n* = 3) were > 0.998, > 0.996, and > 0.997 for abemaciclib, palbociclib, and ribociclib, respectively. The regression equations were *y* = (0.0115 ± 0.0004) *x* + (0.001 ± 0.001) for abemaciclib, *y* = (0.0116 ± 0.0006) *x* + (0.004 ± 0.001) for palbociclib, and *y* = (0.0099 ± 0.0009) *x* + (0.0009 ± 0.0019) for ribociclib, where *y* is the ratio of the analyte and IS response and *x* is the analyte concentration in nanograms per milliliter. All back-calculated concentrations from non-zero calibrators were within ± 15% of the nominal concentrations. Thus, the method was linear in the concentration range of 2–200 ng/mL for each analyte.

#### Selectivity and specificity

All batches of both human and mouse plasma were free of interferences for the analytes and IS at their corresponding mass transitions. Furthermore, the spiked LLOQ human and mouse plasma samples were within ± 20% of the nominal concentration, for at least 4 out of 6 tested batches for all analytes. For tissue homogenates, none of the blanks evaluated showed any interference, and the bias obtained for the LLOQ was within the acceptance criteria.

Moreover, cross analyte and IS did not show any interference, except for ribociclib-IS (RBC-IS) which interfered with ribociclib response. However, the peak response of RBC-IS was 16.1% of the ribociclib LLOQ area. Since this value is ≤ 20%, it was found to be acceptable.

#### Lower limit of quantification

The peak height at the LLOQ was more than 13 times the noise obtained with the blank response in three validation runs for all analytes. Furthermore, the accuracy and precision for each analyte in human plasma were within ± 20% and ≤ 20%, respectively, in three analytical runs (Table 3). For the other matrices, the LLOQ met these acceptance criteria as well (Table 4).

**Table 3:** Accuracy and precision of abemaciclib, palbociclib, and ribociclib in human plasma

	Nominal concentration (ng/mL)	Intra-assay		Inter-assay	
		Bias (%)	CV (%)	Bias (%)	CV (%)
Abemaciclib	2	± 11.4	≤ 11.0	− 7.6	1.8
	5	± 7.2	≤ 5.0	− 5.4	1.1
	50	± 4.6	≤ 4.3	3.7	*
	150	± 3.1	≤ 2.7	2.0	0.4
Palbociclib	2	± 18.5	≤ 10.9	− 9.9	10.9
	5	± 9.6	≤ 8.9	− 8.1	*
	50	± 2.5	≤ 4.8	− 2.0	*
	150	± 6.4	≤ 3.0	− 3.0	3.2
Ribociclib	2	± 18.5	≤ 10.0	− 13.3	4.0
	5	± 13.5	≤ 4.8	− 9.7	4.4
	50	± 6.2	≤ 3.9	− 4.6	2.5
	150	± 5.2	≤ 4.4	− 5.2	3.6

*The inter-run precision cannot be calculated, because the mean square between groups is lower than the mean square within groups (calculated with analysis of variances, ANOVA). This means that there is no significant additional variation that can be assigned to the analysis in different runs

**Table 4:** Accuracy and precision of abemaciclib, palbociclib, and ribociclib inhibitors in mouse plasma and tissue homogenates

Matrix	Nominal concentration (ng/mL)	Abemaciclib		Palbociclib		Ribociclib	
		Bias (%)	CV (%)	Bias (%)	CV (%)	Bias (%)	CV (%)
Plasma	2	− 3.4	4.6	− 4.2	7.0	− 11.2	4.7
	5	− 0.5	4.7	− 8.6	5.2	− 8.7	8.6
	50	3.3	2.0	− 7.5	3.6	− 4.8	3.0
	150	0.5	2.3	− 7.1	2.1	− 8.3	2.6
Liver homogenate	2	14.1	13.2	11.1	9.6	5.0	9.3
	5	10.3	2.8	14.1	7.7	10.0	7.7
	50	14	3.3	14.2	3.5	8.2	2.7
	150	10.7	2.3	18.4	1.9	6.4	1.9
Kidney homogenate	2	12.3	6.5	12.4	2.3	8.9	8.0
	5	8.2	6.6	7.6	7.5	1.2	4.7
	50	9.7	1.8	12.8	1.7	0.4	1.8
	150	9.1	3.4	10.5	3.6	0.3	3.8
Spleen homogenate	2	3.9	8.8	16.4	9.8	4.1	12.6
	5	5.7	5.3	10.3	3.5	0.2	1.1
	50	13.9	2.3	12.4	2.6	6.4	4.1
	150	13.5	2.6	12.7	1.3	8.1	3.0
Brain homogenate	2	6.3	10.4	11.5	7.8	1.6	11.7
	5	8.1	2.6	9.4	2.8	− 3.8	4.5
	50	11.6	3.4	4.6	3.8	6.4	4.1
	150	13.6	1.8	4.5	2.5	1.7	1.4
Small intestine homogenate	2	10.2	6.3	11.9	7.4	18.4	3.7
	5	15.4	6.8	7.7	9.1	12.5	7.2
	50	6.6	2.1	12.2	4.4	8.7	3.8
	150	− 0.1	2.3	8.0	3.7	0.1	2.4

#### Carryover

The carryover was evaluated in three analytical runs and the percentage obtained was ≤ 14.0%, 14.5%, and 6.4% of the LLOQ for abemaciclib, palbociclib, and ribociclib, respectively. The internal standards showed a negligible carryover since it was ≤ 0.4% compared with the mean response of each internal standard.

#### Accuracy and precision

In Table 3, the intra- and inter-assay accuracy and precision results for the quantification of the CDK4/6 inhibitors in human plasma are presented. The bias and CV were within ± 20% and ≤ 20% at the LLOQ, and within ± 15% and ≤ 15% for the other QC samples for all analytes. These results meet the acceptance criteria established in the official FDA and EMA guidelines [15, 16]. For the other matrices, intra-assay accuracy and precision were assessed and the results are shown in Table 4. For mouse plasma, all the data meet the criteria previously mentioned. For the tissue homogenates, the bias was within ± 20% at all concentration levels, and the CV was ≤ 15%. These results meet the criteria for a qualified method according to Xue et al. [17]. Since the QC samples meet the acceptance criteria, the quantification of the three CDK4/6 inhibitors in mouse plasma and tissue homogenates using human plasma as a surrogate matrix is accepted. This involves the use of calibration standards prepared in human plasma to quantify the analytes in the tested matrices.

#### Matrix factor and recovery

The CV of the normalized matrix factor at both concentration levels was ≤ 7.4% for all analytes. The mean normalized matrix factor was between 1.05 and 1.08, between 1.09 and 1.19, and between 1.11 and 1.20, for abemaciclib, palbociclib, and ribociclib, respectively. The matrix showed no significant effect on the analyte responses, since the normalized MF values are close to 1. The absolute matrix factor for each analyte and internal standard are described in the Electronic Supplementary Material (ESM) Table S1.

Furthermore, the normalized recovery after the extraction process was consistent at low and high concentrations for each analyte, 82.8% (CV = 1.6%) for abemaciclib, 79.9% (CV = 5.6%) for palbociclib, and 83.4% (CV = 4.1%) for ribociclib. Absolute recoveries are shown in the ESM Table S2.

#### Dilution integrity

A 1:5 dilution of mouse plasma samples in human plasma was investigated during the validation, since the volume of plasma obtained from a pharmacokinetics experiment in mice is low (10–20 μL). To reach a sample volume of 50 μL necessary for the sample pretreatment, 10 μL of mouse plasma with high concentration of the analytes was diluted with 40 μL of control human plasma. The bias and CV values obtained were within ± 3.7% and ≤ 3.5% for abemaciclib, ± 4.5% and ≤ 7.5% for palbociclib, and ± 9.4% and ≤ 12.9% for ribociclib, complying with the acceptance criteria.

On the other hand, the bias and CV obtained for 10-fold diluted tissue samples with human plasma were ± 7.6% and ≤ 5.4% for abemaciclib, ± 4.0% and ≤ 4.6% for palbociclib, and ± 5.5% and ≤ 4.6% for ribociclib, respectively. All these results meet the acceptance criteria (± 20% and ≤ 20%) for method qualification.

#### Stability

Stock solutions of abemaciclib, palbociclib, and ribociclib were stable at − 20 °C after at least 12 months of storage. Plasma stability, in both mouse and human matrices, is presented in Table 5. In summary, all the analytes were stable in plasma at all stability conditions tested, except for QC L ribociclib after 3 F/T cycles and QC H abemaciclib in mouse plasma samples after 3 months of storage at − 20 °C. Ribociclib QC L showed a mean bias slightly higher than ± 15%, where only one of three samples was outside the acceptance criteria. However, after the second F/T cycle, the mean bias of ribociclib was − 5.4% and the CV was 5.4%, demonstrating that ribociclib is stable in human plasma after 2 F/T cycles. Abemaciclib QC H sample was stable in human plasma for 12 months, but it was not in mouse plasma after 3 months of storage since the bias was > 15%, although there is no indication of degradation since the bias is positive.

**Table 5:** Stability of abemaciclib, palbociclib, and ribociclib in mouse and human plasma

Analyte	Matrix	Stability conditions	Nominal concentration (ng/mL)	Mean concentration (ng/mL)	Accuracy (% Bias)	Precision (% CV)
Abemaciclib	Human plasma	RT, 3 d	5	4.8	− 4.5	6.1
			150	159.3	6.2	1.4
		3 F/T (− 20 °C/RT)	5	4.7	− 6.5	5.2
			150	144.3	− 3.8	1.1
		− 20 °C, 12 m	5	4.8	− 4.7	0.8
			150	141.7	− 5.6	3.5
	Final extract	4–8 °C, 4 d	5	5.2	4.5	2.6
			150	160.7	7.1	1.8
	Mouse plasma	RT, 3 d	5	4.8	− 4.6	4.7
			150	159.3	6.2	1.6
		3 F/T (− 20 °C/RT)	5	5.2	4.3	2.6
			150	161.3	7.6	3.6
		− 20 °C, 3 m	5	5.1	1.0	10.1
			150	182.7	21.8	0.3
	Final extract	4–8 °C, 4 d	5	5.4	8.1	3.2
			150	157.7	5.1	6.4
Palbociclib	Human plasma	RT, 3 d	5	4.8	− 3.8	2.8
			150	142.7	− 4.9	2.1
		3 F/T (− 20 °C/RT)	5	4.3	− 14.5	0.4
			150	137.7	− 8.2	1.1
		− 20 °C, 12 m	5	4.4	− 12.8	1.5
			150	137.0	− 8.7	3.2
	Final extract	4–8 °C, 4 d	5	4.7	− 6.9	5.2
			150	148.7	− 0.9	0.8
	Mouse plasma	RT, 3 d	5	4.5	− 10.1	1.3
			150	141.3	− 5.8	3.2
		3 F/T (− 20 °C/RT)	5	4.6	− 8.8	6.1
			150	149.7	− 0.2	2.7
		− 20 °C, 3 m	5	5.1	1.4	7.3
			150	166.7	11.1	3.0
	Final extract	4–8 °C, 4 d	5	5.0	− 0.8	5.9
			150	142.7	− 4.9	5.3
Ribociclib	Human plasma	RT, 3 d	5	4.6	− 8.9	2.4
			150	144.3	− 3.8	3.3
		3 F/T (− 20 °C/RT)	5	4.3	− 16.3	9.3
			150	136.3	− 9.1	1.8
		2 F/T (− 20 °C/RT)	5	4.7	− 5.4	5.4
		− 20 °C, 12 m	5	4.9	− 2.7	0.5
			150	134.3	− 10.4	0.9
	Final extract	4–8 °C, 4 d	5	4.6	− 7.5	3.9
			150	142.7	− 4.9	4.8
	Mouse plasma	RT, 3 d	5	4.5	− 9.9	2.8
			150	144.0	− 4.0	0.0
		3 F/T (− 20 °C/RT)	5	5.0	− 0.3	3.2
			150	147.7	− 1.6	1.4
		− 20 °C, 3 m	5	4.8	− 3.7	5.5
			150	157.0	4.7	0.6
	Final extract	4–8 °C, 4 d	5	4.8	− 4.5	3.8
			150	141.0	− 6.0	3.5

*RT* room temperature, *F/T* freeze-thaw cycles, *d* days, *m* months

The stability in tissue homogenates is reported in Table 6. It shows that abemaciclib is stable in all matrices at all conditions. In contrast, palbociclib and ribociclib showed instability in tissue homogenates. For example, palbociclib or ribociclib were not stable in the liver, kidney, and small intestine when stored at − 20 °C for 1 month; therefore, the long-term stability was tested at − 70 °C where both compounds were stable in all matrices. After 24 h at room temperature, palbociclib and ribociclib showed significant degradation in the kidney and spleen homogenates; however, they were stable for at least 3 h at room temperature and in an ice/water bath. Thus, the instability of these two analytes in the kidneys after freeze/thaw cycles from − 20 °C to room temperature is likely a consequence of their instability at the previously described conditions. Therefore, the stability after 3 freeze/thaw cycles from − 70 °C to ice/water bath was tested, and at these conditions, the analytes were found to be stable. In spite of the instability of palbociclib and ribociclib in some tissue homogenates, appropriate conditions for samples storage and processing have been found.

**Table 6:** Stability of abemaciclib, palbociclib, and ribociclib in tissue homogenates

Analyte	Matrix	Stability conditions	Nominal concentration (ng/mL)	Mean concentration (ng/mL)	Accuracy (% Bias)	Precision (% CV)
Abemaciclib	Liver homogenate	RT, 1 d	50	47.9	− 4.1	1.4
		Ice/water bath, 3 h	50	51.9	3.8	3.0
		3 F/T (RT, − 20 °C)	50	52.5	5.0	5.8
		3 F/T (− 70 °C, ice/water bath)	50	50.0	0.1	1.3
		LT − 20 °C, 1 m	50	50.7	1.4	5.3
		LT − 70 °C, 1 m	50	50.6	1.2	3.4
	Final extract	4–8 °C, 2 d	50	47.9	− 4.1	8.0
	Spleen homogenate	RT, 1 d	50	46.5	− 6.9	2.9
		RT, 3 h	50	51.7	3.4	4.7
		Ice/water bath, 3 h	50	52.4	4.9	9.3
		3 F/T (RT, − 20 °C)	50	48.7	− 2.6	4.7
		3 F/T (− 70 °C, ice/water bath)	50	56.1	12.1	3.5
		LT − 20 °C, 1 m	50	48.6	− 2.7	3.0
		LT − 70 °C, 1 m	50	51.8	3.5	2.3
	Final extract	4–8 °C, 3 d	5	5.5	10.7	4.2
			150	156.3	4.2	10.7
	Kidney homogenate	RT, 1 d	50	43.3	− 13.5	6.7
		RT, 3 h	50	50.9	1.7	0.6
		Ice/water bath, 3 h	50	49.3	− 1.3	1.4
		3 F/T (RT, − 20 °C)	50	49.5	− 1.1	12.1
		3 F/T (− 70 °C, ice/water bath)	50	51.8	3.6	6.3
		LT − 20 °C, 1 m	50	49.0	− 1.9	3.9
		LT − 70 °C, 1 m	50	52.7	5.5	0.8
	Final extract	4–8 °C, 2 d	50	48.8	− 2.3	1.4
	Brain homogenate	RT, 1 d	5	5.3	6.2	3.0
			150	167	11.3	9.4
		Ice/water bath, 3 h	50	55.2	10.4	1.6
		3 F/T (RT, − 20 °C)	50	53.4	6.9	3.0
		3 F/T (− 70 °C, ice/water bath)	50	51.1	2.2	6.2
		LT − 20 °C, 1 m	50	52.0	3.9	4.7
		LT − 70 °C, 1 m	50	52.1	4.3	5.8
	Final extract	4–8 °C, 3 d	5	4.9	− 1.5	8.0
			150	163	8.7	2.2
	Small intestine homogenate	RT, 1 d	50	46.4	− 7.1	1.3
		RT, 3 h	50	56.4	12.3	1.5
		Ice/water bath, 3 h	50	51.6	3.1	3.5
		3 F/T (RT, − 20 °C)	50	49.0	− 2.0	4.2
		3 F/T (− 70 °C, ice/water bath)	50	50.7	1.5	5.3
		LT − 20 °C, 1 m	50	48.2	− 3.7	2.2
		LT − 70 °C, 1 m	50	48.3	− 3.4	4.0
	Final extract	4–8 °C, 2 d	50	52.4	4.7	6.1
Palbociclib	Liver homogenate	RT, 1 d	50	44.9	− 10.2	4.8
		Ice/water bath, 3 h	50	49.8	− 0.5	5.2
		3 F/T (RT, − 20 °C)	50	43.6	− 12.7	2.7
		3 F/T (− 70 °C, ice/water bath)	50	50.6	1.2	1.5
		LT − 20 °C, 1 m	50	34.3	− 31.5	6.7
		LT − 70 °C, 1 m	50	47.1	− 1.9	5.8
	Final extract	4–8 °C, 2 d	50	41.3	− 17.4	4.2
	Spleen homogenate	RT, 1 d	50	30.7	− 38.5	6.0
		RT, 3 h	50	47.2	− 5.7	8.4
		Ice/water bath, 3 h	50	46.0	− 8.1	5.8
		3 F/T (RT, − 20 °C)	50	42.1	− 15.8	3.8
		3 F/T (− 70 °C, ice/water bath)	50	47.9	− 4.2	1.3
		LT − 20 °C, 1 m	50	42.6	− 14.9	3.8
		LT − 70 °C, 1 m	50	46.9	− 6.1	4.1
	Final extract	4–8 °C, 3 d	5	4.9	− 1.3	5.7
			150	157	4.7	6.3
	Kidney homogenate	RT, 1 d	50	2.91	− 94.2	11.8
		RT, 3 h	50	45.3	− 9.4	13.6
		Ice/water bath, 3 h	50	43.4	− 13.3	6.6
		3 F/T (RT, − 20 °C)	50	19.3	− 61.5	19.5
		3 F/T (− 70 °C, ice/water bath)	50	52.0	4.1	0.7
		LT − 20 °C, 1 m	50	33.2	− 33.6	7.9
		LT − 70 °C, 1 m	50	47.9	− 4.1	3.3
	Final extract	4–8 °C, 2 d	50	46.5	− 7.1	2.6
	Brain homogenate	RT, 1 d	5	4.7	− 6.5	8.4
			150	149.3	− 0.4	7.2
		Ice/water bath, 3 h	50	49.4	− 1.2	3.1
		3 F/T (RT, − 20 °C)	50	47.3	− 5.3	5.8
		3 F/T (− 70 °C, ice/water bath)	50	51.0	2.1	1.9
		LT − 20 °C, 1 m	50	46.7	− 6.6	2.1
		LT − 70 °C, 1 m	50	52.1	4.2	1.4
	Final extract	4–8 °C, 3 d	5	5.4	7.5	0.9
			150	168.3	12.2	2.4
	Small intestine	RT, 1 d	50	43.8	12.5	1.5
		RT, 3 d	50	48.5	− 3.0	7.0
		Ice/water bath, 3 h	50	49.0	− 2.0	9.6
		3 F/T (RT, − 20 °C)	50	44.6	− 10.7	3.5
		3 F/T (− 70 °C, ice/water bath)	50	49.9	− 0.3	3.6
		LT − 20 °C, 1 m	50	42.2	− 15.5	7.2
		LT − 70 °C, 1 m	50	48.8	− 2.3	3.7
	Final extract	4–8 °C, 2 d	50	45.9	− 8.2	5.9
Ribociclib	Liver homogenate	RT, 1 d	50	41.9	− 16.2	7.3
		Ice/water bath, 3 h	50	45.4	− 9.3	4.7
		3 F/T (RT, − 20 °C)	50	47.7	− 4.7	2.7
		3 F/T (− 70 °C, ice/water bath)	50	50.0	0.1	1.7
		LT − 20 °C, 1 m	50	32.4	− 35.1	13.9
		LT − 70 °C, 1 m	50	49.1	− 1.9	7.2
	Final extract	4–8 °C, 2 d	50	42.6	− 14.9	6.6
	Spleen homogenate	RT, 1 d	50	21.8	− 56.4	6.4
		RT, 3 h	50	44.3	− 11.5	5.8
		Ice/water bath, 3 h	50	46.2	− 7.5	2.3
		3 F/T (RT, − 20 °C)	50	42.9	− 14.1	1.2
		3 F/T (− 70 °C, ice/water bath)	50	49.9	− 0.1	1.4
		LT − 20 °C, 1 m	50	40.5	− 19.1	4.3
		LT − 70 °C, 1 m	50	45.0	− 9.9	1.2
	Final extract	4–8 °C, 3 d	5	5.16	3.2	4.1
			150	148.7	− 0.9	0.8
	Kidney homogenate	RT, 1 d	50	0.91	− 98.2	24.4
		RT, 3 h	50	43.4	− 13.2	1.2
		Ice/water bath, 3 h	50	42.7	− 14.6	1.5
		3 F/T (RT, − 20 °C)	50	15.7	− 68.6	30.1
		3 F/T (− 70 °C, ice/water bath)	50	49.1	− 1.7	3.4
		LT − 20 °C, 1 m	50	31.5	− 37.1	4.1
		LT − 70 °C, 1 m	50	50.2	0.5	3.6
	Final extract	4–8 °C, 2 d	50	43.2	− 13.5	6.7
	Brain homogenate	RT, 1 d	5	4.6	− 7.9	1.8
			150	129.7	− 13.6	5.1
		Ice/water bath, 3 h	50	49.4	− 1.3	4.3
		3 F/T (RT, − 20 °C)	50	48.8	− 2.3	5.8
		3 F/T (− 70 °C, ice/water bath)	50	50.0	0.1	2.1
		LT − 20 °C, 1 m	50	49.1	− 1.7	2.0
		LT − 70 °C, 1 m	50	52.3	4.6	4.7
	Final extract	4–8 °C, 3 d	5	5.1	2.7	5.1
			150	154.7	3.1	1.9
	Small intestine homogenate	RT, 1 day	50	41.9	− 16.3	3.2
		RT, 3 h	50	50.6	1.3	3.5
		Ice/water bath, 3 h	50	48.4	− 3.2	5.3
		3 F/T (RT, − 20 °C)	50	45.6	− 8.9	0.3
		3 F/T (− 70 °C, ice/water bath)	50	50.0	0.1	3.6
		LT − 20 °C, 1 m	50	38.9	− 22.2	10.5
		LT − 70 °C, 1 m	50	48.9	− 2.1	2.6
	Final extract	4–8 °C, 2 d	50	44.2	− 11.5	7.3

*RT* room temperature, *F/T* freeze-thaw cycles, *LT* long term, *d* days, *m* months

### Preclinical application of the method

**Fig. 3:**
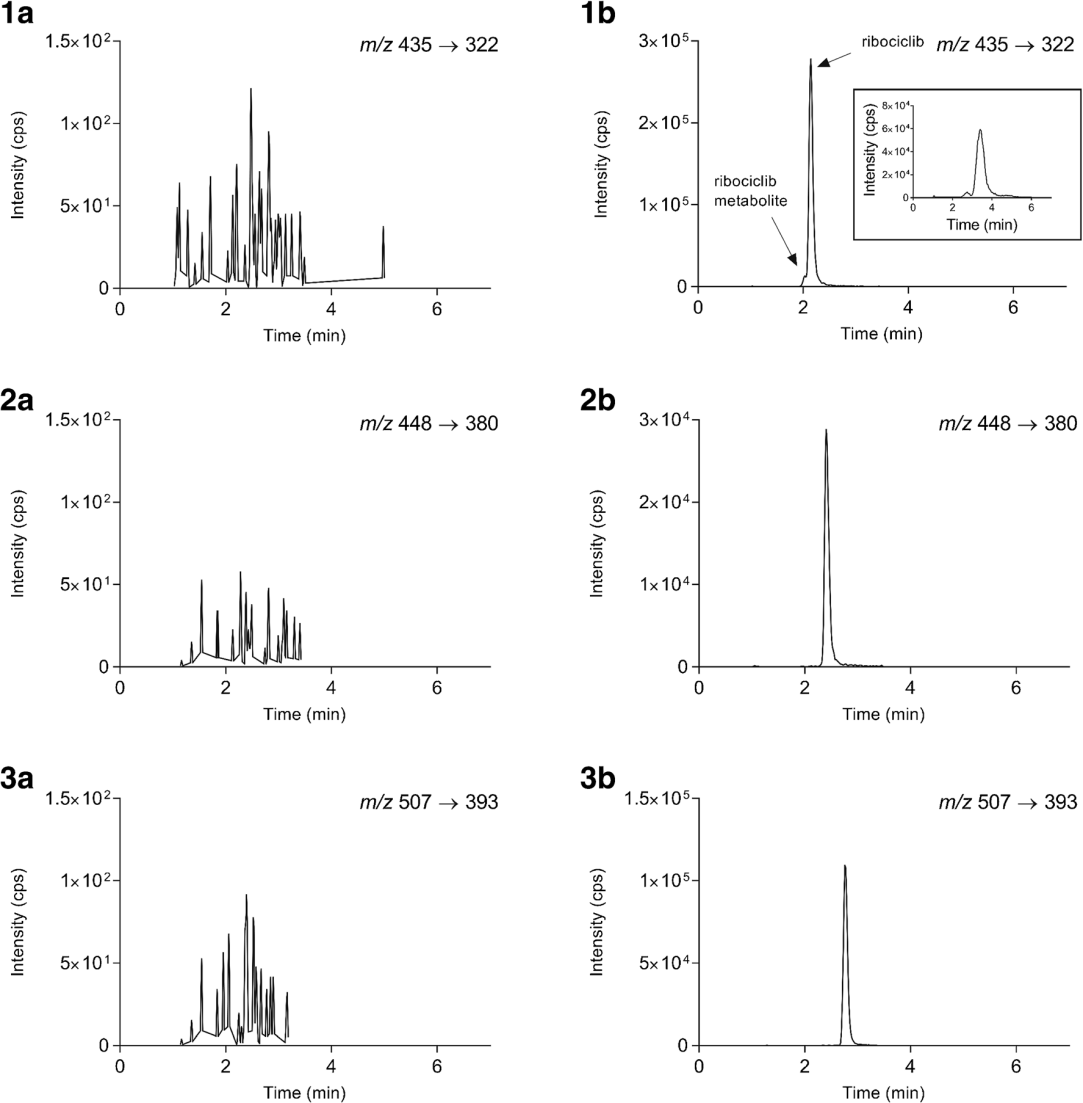
Chromatograms of blank mouse plasma at the mass transitions of ribociclib (**a**), palbociclib (**b**), and abemaciclib (**c**), and representative chromatograms of samples from preclinical studies of ribociclib (**d**, 20 mg/kg orally administered, *t* = 8 h, 625 ng/mL), palbociclib (**e**, 10 mg/kg orally administered, *t* = 2 h, 311 ng/mL), and abemaciclib (**f**, 10 mg/kg orally administered, *t* = 24 h, 74.3 ng/mL). The additional figure included in **d** shows the same acquired with different gradient to increase the resolution between the two peaks. The figure was created using the output form Sciex Analyst software along with GraphPad Prism 7 software

This method was successfully used for the analysis of samples from preclinical studies of the CDK4/6 inhibitors. Figure 3 depicts a representative chromatogram from a preclinical study in mice for each drug. As shown in panel d, the chromatogram from a mouse plasma sample contains an extra peak eluting prior to ribociclib. This unknown peak was only present in mouse plasma samples and not in spiked samples or tissue homogenates, which indicates it is a potential ribociclib metabolite. In order to obtain the HR-MS spectra from each peak, the gradient was modified to increase the resolution between ribociclib and the potential metabolite (Fig. 3, additional figure in panel d). The analysis generated a mass spectrum of both peaks, and the accurate mass of ribociclib was 435.26489 Da (mass difference of 5.58 ppm from the theoretical accurate monoisotopic mass), and for the compound eluting just before ribociclib, a mass of 435.22830 Da was found (ESM Fig. S1). The MS spectrum of the unknown peak was analyzed with the software Compound Discoverer 2.1.0401 (Thermo Fisher Scientific) and the proposed molecular formula is C_22_H_26_N_8_O_2_. When the molecular formula of this potential metabolite is compared with the parent drug (C_23_H_30_N_8_O), N-desmethylation, oxidation, and reduction of the ribociclib molecule are suggested as metabolic conversions. To identify the metabolite, further research is needed. A semi-quantification was performed using ribociclib calibration standards and the metabolite concentration was less than 10% of ribociclib concentration in each sample. For this reason, it was not considered as a major metabolite.

**Fig. 4:**
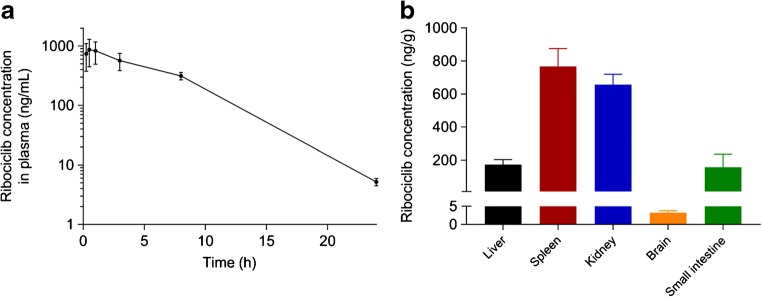
Plasma concentration-time curve (**a**) and tissue concentration (**b**) of ribociclib over 24 h in female FVB mice after oral administration at 20 mg/kg. Data are presented as mean ± SD (*n* = 6). The figure was created using the GraphPad Prism 7 software

As an example of the preclinical pharmacokinetics and tissue distribution study, the plasma concentration-time plasma curve of ribociclib is shown in Fig. 4a. The calculated pharmacokinetic parameters of ribociclib were the AUC_0–24h_ of 6885 ± 1215 ng mL^−1^ h, the half-life is 3.1 ± 0.2 h, the *T*_max_ range is from 0.5–1 h, in which the mean *C*_max_ is 1036 ± 352 ng/mL. Ribociclib was also quantified in tissue homogenates, and drug concentration in tissues was calculated considering the total weight of each organ. Ribociclib was highly distributed in the spleen and kidney, followed by the liver and small intestine; in the brain, the concentration of ribociclib was much lower compared with that in the other tissues (Fig. 4b). In this analysis, 95% of the samples were within the validated range showing the applicability of the method to preclinical studies.

## Conclusion

The first validated multi-assay of the first three approved CDK4/6 inhibitors (abemaciclib, palbociclib, and ribociclib) in plasma and tissue homogenates has been described. Human plasma can be used as a surrogate matrix for the quantification of these compounds in mouse plasma and tissue homogenates. As palbociclib and ribociclib showed instability in some tissue homogenates, it is recommended to store these samples at − 70 °C and process them within 3 h when these samples are kept at room temperature. This method can be used for both preclinical and clinical studies. In this article, this method was successfully applied to a preclinical study of ribociclib in mice. A ribociclib metabolite was detected in mouse plasma samples with the same *m/z* transition as the parent drug, which was not previously described.

## Electronic supplementary material

ESM 1 (PDF 130 kb)
