# Supplementary material for: Development and validation of a bioanalytical method for the quantification of the CDK4/6 inhibitors abemaciclib, palbociclib, and ribociclib in human and mouse matrices using liquid chromatography-tandem mass spectrometry
Source: Anal Bioanal Chem. 2019 Jun 17;411(20):5331–45. doi: 10.1007/s00216-019-01932-w (PMC6647725; doi:10.1007/s00216-019-01932-w)
Supplement: Supplementary file 1 — (PDF 130 kb) [file 216_2019_1932_MOESM1_ESM.pdf]

Analytical and Bioanalytical Chemistry

Electronic Supplementary Material

Development and validation of a bioanalytical method for the quantification of the CDK4/6 inhibitors abemaciclib, palbociclib and ribociclib in human and mouse matrices using liquid chromatography-tandem mass spectrometry

Alejandra Martínez-Chávez, Hilde Rosing, Michel Hillebrand, Matthijs Tibben, Alfred H. Schinkel, Jos H. Beijnen

Table S1 Absolute and normalized matrix factor of abemaciclib, palbociclib and ribociclib at high and low concentrations

	Concentration level	Analyte	IS	Normalized
Abemaciclib	Low	1.022 ± 0.026	0.976 ± 0.059	1.052 ± 0.077
	High	1.107 ± 0.027	1.025 ± 0.028	1.081 ± 0.030
Palbociclib	Low	1.071 ± 0.053	0.903 ± 0.042	1.188 ± 0.071
	High	1.034 ± 0.027	0.940 ± 0.020	1.100 ± 0.024
Ribociclib	Low	1.195 ± 0.082	0.993 ± 0.020	1.203 ± 0.072
	High	1.078 ± 0.017	0.972 ± 0.034	1.110 ± 0.031

Data are presented as mean ± SD (n = 6)

Table S2 Absolute and normalized recovery (%) of abemaciclib, palbociclib and ribociclib at high and low concentrations

	Concentration level	Analyte	IS	Normalized	Mean recovery (%CV)
Abemaciclib	Low	63.2	77.0	81.9	82.8 (1.6)
	High	60.0	71.7	83.7	
Palbociclib	Low	75.1	97.6	76.8	79.9 (5.6)
	High	81.9	98.9	83.0	
Ribociclib	Low	76.5	94.4	80.9	83.4 (4.1)
	High	77.7	90.3	85.8	

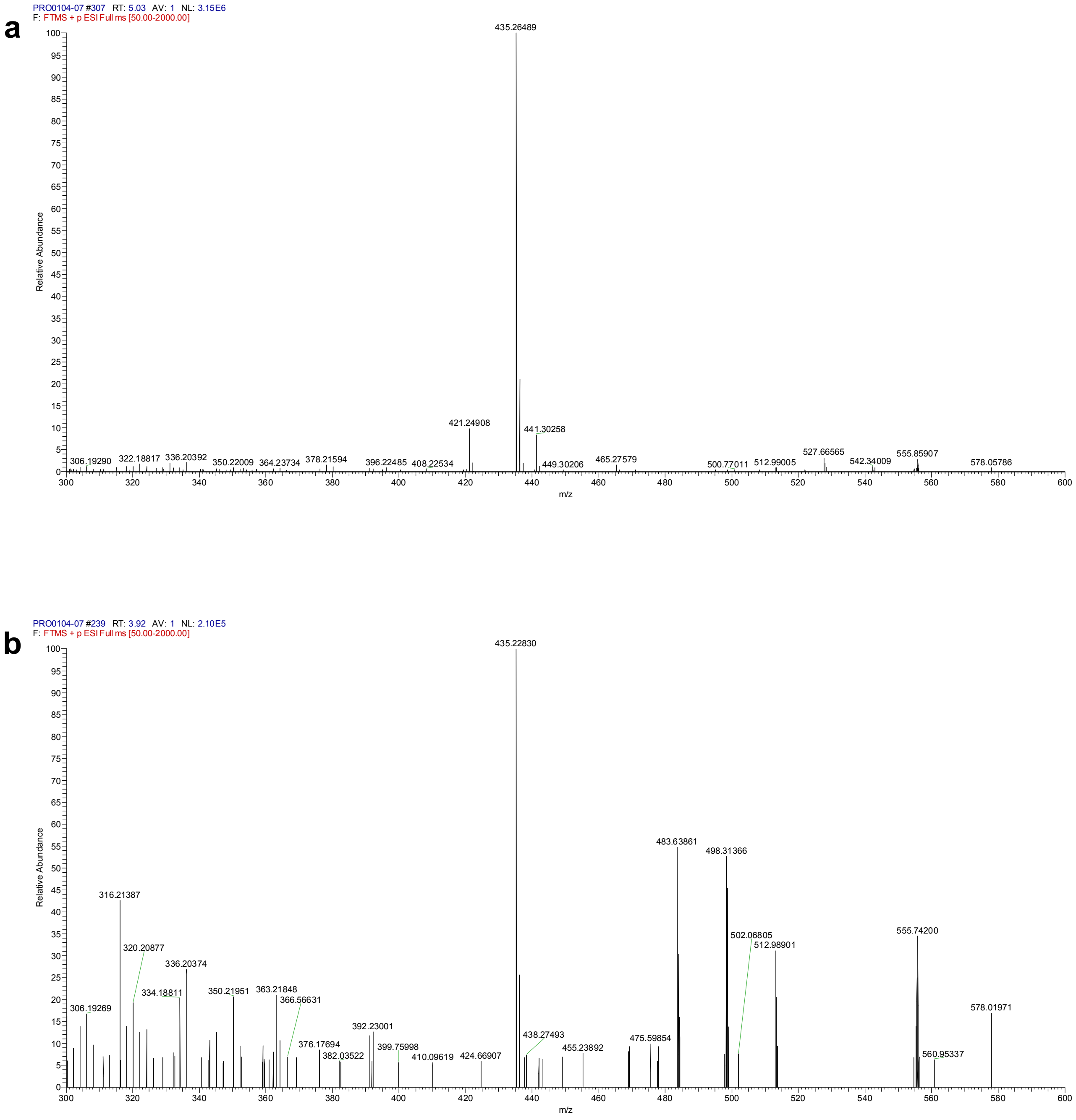

Fig. S1 Mass spectrum of the parent ion of ribociclib (a) and potential ribociclib metabolite (b)
